# The Role of Estrogen Receptors in Urothelial Cancer

**DOI:** 10.3389/fendo.2021.643870

**Published:** 2021-03-16

**Authors:** Takuro Goto, Hiroshi Miyamoto

**Affiliations:** ^1^ Department of Pathology and Laboratory Medicine, University of Rochester Medical Center, Rochester, NY, United States; ^2^ James P. Wilmot Cancer Institute, University of Rochester Medical Center, Rochester, NY, United States; ^3^ Department of Urology, University of Rochester Medical Center, Rochester, NY, United States

**Keywords:** bladder cancer, estrogen receptor-α, estrogen receptor-β, cancer progression, carcinogenesis, chemoresistance, urothelial cancer

## Abstract

Epidemiological data have indicated that there are some sex-related differences in bladder cancer. Indeed, the incidence of bladder cancer in men has been substantially higher than that in women throughout the world, while women tend to have higher stage disease and poorer prognosis. These gender disparities have prompted to investigate sex hormones and their cognitive receptors in bladder cancer. Specifically, estrogen receptors, including estrogen receptor-α and estrogen receptor-β, have been shown to contribute to urothelial carcinogenesis and cancer progression, as well as to modulating chemosensitivity in bladder cancer, although conflicting findings exist. Meanwhile, immunohistochemical studies in surgical specimens have assessed the expression of estrogen receptors and related proteins as well as its associations with clinicopathologic features of bladder cancer and patient outcomes. This review article summarizes and discusses available data indicating that estrogen receptor signaling plays an important role in urothelial cancer.

## Introduction

Urinary bladder cancer, mostly a urothelial carcinoma, has been one of the most commonly diagnosed malignancies (1). In addition to the bladder (and urethra), 5-10% of urothelial carcinomas occur in the upper urinary tract composed of the renal calyces, renal pelvis, and ureter in 17% of which concurrent bladder cancer is reported to be present (2). The incidence of bladder cancer, as well as upper urinary tract cancer, is known to be 3-4 times higher in men than in women, while the mortality rate among female patients is even higher (1, 3). Importantly, even after various factors, such as exposure to cigarettes and occupational hazards, have been controlled, the sex-related differences persist (1, 3). Potential delays in the diagnosis of bladder cancer in women, as well as some differences in the treatment practices and anatomy of the bladder between men and women, may still contribute to the gender disparities. For instance, a systematic review showed that women waited longer for being referred to urology clinics and having image studies and/or cystoscopy (4). Meta-analyses also indicated that, compared with male patients, women were less sensitive to intravesical bacillus Calmette-Guérin (BCG) immunotherapy (5), had a significantly higher risk for disease recurrence after transurethral resection of non-muscle-invasive tumors (5), and showed worse disease-free/cancer-specific/overall survival after radical cystectomy for muscle-invasive disease (6). In addition, the prognosis of pT4 disease is significantly worse in women than in men, presumably due to a thinner wall thickness in the female bladders and a difference in the definition of pT4 classification based on gender-specific anatomy (7). Recently, genetic factors have been suggested to contribute to worse outcomes in female patients. Specifically, of the 5 molecular subtypes of bladder cancers defined in The Cancer Genome Atlas cohort, the basal-squamous subtype, which is associated with poor prognosis, is more commonly seen in women than in men (33% vs. 21%; *P* = 0.024) (8).

In addition to those described above, sex hormone receptors, including androgen receptor and estrogen receptors (ERs), have been explored as key intrinsic factors for better understanding the sex-specific differences in bladder cancer. Indeed, androgen receptor activation has been implicated in the induction of urothelial tumorigenesis, which may clearly explain the male dominance in the incidence of bladder cancer, as well as tumor progression (reviewed in 9, 10). Androgen deprivation, often used for the treatment of, for example, prostate cancer, is thus expected to show a benefit in patients with bladder cancer. By contrast, conflicting results exist regarding the relationship between ER activity and urothelial cancer outgrowth. Importantly, molecular mechanisms underlying the actions of these hormone receptors in urothelial cancer cells have not been fully uncovered.

The presence of ER, which is now called ERα, was first demonstrated by Elwood Jensen in 1958 (11), whereas ERβ in rat (12) or human (13) was cloned in 1996 or 1997, respectively. ERα and ERβ are physiologically expressed in various human organs and, upon binding of estrogens such as 17β-estradiol (E2), possess a variety of actions in these tissues (14). In preclinical models for several types of endocrine malignancies, such as breast, ovary, and prostate carcinomas, ERα and ERβ have also been shown to function differently. Additionally, there is an increasing amount of evidence to suggest the involvement of estrogen-mediated ER signaling in the development and progression of urothelial cancer. ER activation has also been associated with one of the molecular subtypes, luminal subtype, in muscle-invasive bladder cancer (15).

We first performed a computerized bibliographic search of the PubMed database, using the following keywords variably combined: “antiestrogen”, “bladder”, “bladder cancer”, “bladder tumor”, “bladder tumour”, “estrogen”, “estrogen receptor”, “urothelial”, “urothelial cancer”, “urothelial tumor”, “urothelial tumour”, and “urothelium”. We then selected only studies published in peer-reviewed journals (plus some articles found in their reference lists). We thus summarized available data on ERα/ERβ expression in surgical specimens, estrogen/ER functions in benign and malignant urothelial cells demonstrated using preclinical models, and clinical trials involving the modulation of ER signaling.

## Expression of ER in Surgical Specimens

The expression of ERα and ERβ has been immunohistochemically investigated in surgical specimens of urothelial tumors in the bladder or the upper urinary tract (16–38). **Tables 1** and **2** summarize the findings from these studies in bladder and upper urinary tract tissues, respectively, which have compared the levels of ERα/ERβ expression in non-neoplastic urothelial tissues vs. urothelial tumors, male vs. female tumors, low-grade vs. high-grade tumors, and/or non-muscle-invasive/≤pT1 vs. muscle-invasive/≥pT2 tumors. In some of the studies, the prognostic significance of ERα/ERβ expression in urothelial tumors was also assessed.

**Table 1:** Immunohistochemical studies on the expression of ERα and ERβ in bladder cancer tissues.

Author, year [reference]	N	Location	Antibody used	Tissue			Gender			Tumor grade			Tumor stage			Prognostic significance
				Non-tumor	Tumor	*P* value	M	F	*P* value	LG	HG	*P* value	NMI	MI	*P* value	
**ERα**																
Kaufmann, 1998 (16)	185	Bladder	6F11 (Novocastra)		18%		14%	22%	0.253†	33% (G1-2)	19% (G3)	0.507	13%	27%	0.004	NA
Basakci, 2002 (17)	121	Bladder	1D5 (Dako)	10%	12%	0.73	10%	23%	NS	19% (G1-2)	44% (G3)	0.01	29%			PFS (NS) CSS (NS) OS (NS)
Croft, 2005 (18)	92	Bladder	6F11 (Ventana)		11-22%‡					6-12%‡ (G1-2)	17-33%‡ (G3)	0.021-0.177†‡	5-9%‡ (Ta)	16-33%‡ (≥T1)	0.010-0.098†‡	NA
Shen, 2006 (19)	224	Bladder	6F11 (Novocastra)		0.9%											NA
Bolenz, 2009 (20)	198	Bladder	1D5 (Dako)		4.5%		4.8%	4.5%	1.000						0.004 (OC > non-OC)	RFS (NS) CSS (NS)
Wei, 2009 (21)	20	Bladder	6F11		0%											NA
Miyamoto, 2012 (22)	188	Bladder	E115 (Epitomics)	50%	27%	<0.001	28%	25%	0.842	38%	23%	0.048	35%	19%	0.014	RPS&PFS/NMI (NS) PFS/MI (NS)
Mashhadi, 2014 (23)	120	Bladder	1D5 (Dako)	1.5%	2.5%	0.67										NA
Tan, 2015 (24)	317	Bladder	1D5 (Dako)		3.8%							NS			NS	CSS (NS)
Tian, 2015 (25)	306	Bladder	6F11 (Leica)		13%											NA
Izumi, 2016 (26)	72*	Bladder	E115 (Epitomics)	57%	31%	0.00615†	31%			36% (G1-2)	22% (G3)	0.383†	31%			RFS (NS)
Imai, 2019 (27)	125 (UC:100)	Bladder	6F11 (Novocastra)		38%		37%	50%	0.347	32%	45%	0.143	32%	50%	0.056	NA
Bernardo, 2020 (28)	80	Bladder	6F11	12%	18%	0.444	18%	11%	0.592	17%	18%	0.934	18%	18%	1.000†	OS (NS)
**ERβ**																
Shen, 2006 (19)	224	Bladder	MYEB		63%					58% (G1-2)	70% (G3)	0.085	54%	80%	<0.001	NA
Kontos, 2010 (29)	140	Bladder	14C8 (Abcam)	93%	76%	0.041†				95% (G1-2)	56% (G3)	<0.001†	83% (T1)	54%	0.011†	NA
Tuygun, 2011 (30)	139	Bladder	EMR02 (Novocastra)	0% (M) 36% (F)	27-30%‡	<0.001† (M+F vs Tumor)	33%	24%	0.402	22-26%‡	31-34%‡	0.44-0.59‡	24-26%‡	36-42%‡	0.16-0.24‡	RFS (NS) PFS (NS)
Han, 2012 (31)	42	Bladder	Not specified (Boster)		81%		86.67%	66.67%	0.1954	86% (G1-2)	50% (G3)	0.0717	80%			RFS (*P*=0.0208)
Miyamoto, 2012 (22)	188	Bladder	14C8 (Abcam)	89%	49%	<0.001	53%	38%	0.109	29%	58%	<0.001	34%	67%	<0.001	PFS/LG (*P*=0.0005)
																PFS/HGNMI (*P*=0.002)
																CSS/MI (*P*=0.0073)
Kauffman, 2013 (32)	72	Bladder	14C8 (Novus)			<0.001 (N<T)									NS	RFS (*P*=0.030) CSS (*P*=0.0018) OS (*P*=0.0061)
Nam, 2014 (33)	169	Bladder	EMR02 (Novocastra)		31%		31%	31%	1.000	27%	41%	0.043	22% (Ta) 42% (T1)		0.004 (Ta vs T1)	RFS (*P*=0.004) PFS (*P*=0.014)
Tan, 2015 (24)	313	Bladder	PPG5/10 (Thermo Scientific)		100%					100%	100%	NS	100%	100%	NS	CSS (*P*=0.055/0.087)‡
Izumi, 2016 (26)	72*	Bladder	14C8 (Abcam)	81%	54%	0.00463†	54%			52% (G1-2)	56% (G3)	1.000†	54%			RFS (NS)
Nguyen, 2017 (34)	30	Bladder	rabbit polyclonal (Santa Cruz Biotechnology)		60%		63%	55%	0.7	33%	67%	0.2	27%	79%	0.07	RFS (NS) CSS (NS)
Bernardo, 2020 (28)	80	Bladder	14C8	83%	91%	0.235†	91%	8%	0.581	92%	91%	1.000	90%	92%	1.000	NA
Goto, 2020 (35)	55	Bladder	H-150 (Santa Cruz Biotechnology)		56%		56%	60%	1.000†		56%			56%		NA

ER, estrogen receptor; UC, urothelial carcinoma; NA, not available; M, male; F, female; NA, not analyzed; LG, low grade; HG, high grade; NMI, non-muscle-invasive; MI, muscle-invasive; OC, organ-confined; RFS, recurrence-free survival; PFS, progression-free survival; CSS, cancer-specific survival; OS, overall survival; NS, not significant.

^†^We calculated two-tailed P values, using Fisher’s exact test.

^‡^Two criteria were used.

*Patients subsequently received androgen deprivation therapy for their prostate cancer.

**Table 2:** Immunohistochemical studies on the expression of ERα and ERβ in upper urinary tract urothelial cancer tissues.

Author, year [reference]	N	Antibody used	Tissue			Gender			Tumor grade			Tumor stage			Prognostic significance
			Non-tumor	Tumor	*P* value	M	F	*P* value	LG	HG	*P* value	≤pT1	≥pT2	*P* value	
**ERα**															
Kashiwagi, 2016 (36)	99	6F11 (Ventana)	40%	18%	0.001	17%	21%	0.790	27%	17%	0.465	11%	23%	0.183	PFS (NS) CSS (NS)
**ERβ**															
Shyr, 2013 (37)	83	NCL-ER-beta (Novocastra)		43%					44%	43%	1.000†	51%	47%	0.815†	NA
Kashiwagi, 2016 (36)	99	H-150 (Santa Cruz Biotechnology)	85%	63%	0.001	58%	69%	0.296	73%	61%	0.402	65%	63%	1.000	PFS (NS) CSS (NS)
Luo, 2016 (38)	105	mouse monoclonal		50%						50%			50%		Local recurrence (*P*=0.035) Distant metastasis (*P*=0.004)

ER, estrogen receptor; NA, not available; M, male; F, female; NA, not analyzed; LG, low grade; HG, high grade; PFS, progression-free survival; CSS, cancer-specific survival; NS, not significant.

^†^We calculated two-tailed P values, using Fisher’s exact test.

The positive rate of ERα expression in urothelial tumors ranged from 0% to 38% (16–28, 36). ERα expression was shown to be significantly down-regulated in tumors, compared with non-tumors, in three studies (22, 26, 36), while the positivity was even lower, with no statistical significance, in non-tumors in other three studies (17, 23, 28). None of the studies where ERα expression was compared between male and female tumors showed significant differences (16, 17, 20, 22, 27, 28, 36). Two studies (17, 18) showed significant up-regulation of ERα expression in grade 3 tumors (vs. grade 1-2 tumors), whereas the down-regulation in high-grade tumors was reported in at least four of the other studies (no statistical significance in three of them) (16, 22, 26, 36). Similarly, compared with lower stage tumors, both significant (16, 18) or marginal (27) up-regulation and significant down-regulation (20, 22) in higher stage tumors, such as muscle-invasive bladder cancer, were observed. However, there were no significant associations of ERα expression with patient outcomes (17, 20, 22, 24, 26, 28, 36).

ERβ expression has been reported to be positive in 27-100% of urothelial tumors (19, 22, 24, 26, 28–31, 33–38). Compared with non-neoplastic urothelial tissues, the positivity of ERβ expression in tumors was significantly lower in four studies (22, 26, 29, 36) and significantly higher in two studies (30, 32). However, no studies demonstrated significant gender differences in ERβ expression in tumors (22, 28, 30, 31, 33–36). Significant up-regulation (22, 33) or down-regulation (29) in high-grade or grade 3 tumors was observed, while other studies (19, 24, 26, 28, 30, 31, 34, 36, 37) failed to show significant differences between low-grade and high-grade tumors. Similarly, significant up-regulation (19, 22, 33) and down-regulation (29) in higher stage tumors were seen only in some of the studies. In addition, ERβ expression was strongly associated with worse (22, 30, 32, 33) or better (31, 38) outcomes, while no prognostic values of ERβ status were found in other studies (24, 26, 30, 34, 36).

The levels of *ERα* mRNA expression have also been determined in bladder tumor tissues. In these studies, considerable increases in *ERα* expression were found in tumors (vs. normal-appearing bladder tissues) (39) or higher grade/stage tumors (40), and its elevation in muscle-invasive tumors (showing low androgen receptor expression) was associated with the risk of disease progression after radical cystectomy (41). However, three independent databases showed the reduction of *ERα* gene expression in bladder cancer (42).

Inconsistent data on ERα and ERβ expression in urothelial tumor samples have thus been reported, which makes difficult to infer whether ERα/ERβ signals promote or inhibit tumor outgrowth. These discrepancies in immunohistochemical studies may have been attributed to the use of different antibodies and/or protocols for staining as well as the lack of standardization in scoring. Remarkably, significant questions have been raised regarding the specificity of commercially available ER antibodies (43, 44). In particular, only two (*i.e.* PPZ0506, 14C8) of 13 commercially available anti-ERβ antibodies were shown to specifically target ERβ in immunohistochemical staining, while in immunoblotting some of these, including 14C8, preferentially targeted other nuclear protein(s) over ERβ (43). More problematically, PPG5/10 was found not to target ERβ (43, 44). Therefore, for instance, a study, using PPG5/10 while showing no negative cases in 313 bladder tumors (24), might not be creditable. Additionally, because it is well known that delay to formalin fixation after specimen collection leads to false-negative results in ER staining in, for example, breast tissues (45), differences in tissue preparation including preservation in fixative among studies may have affected the immunoreactivity. A meta-analysis of immunohistochemical studies performed in 2017 showed the significant down-regulation of ERα expression in bladder tumors as well as the significant up-regulation of ERβ expression in high-grade or muscle-invasive tumors (46). ERβ positivity in non-muscle-invasive tumors was also found to associate with a higher risk of disease recurrence (hazard ratio = 1.573; *P* = 0.013) or progression (hazard ratio = 2.236; *P* = 0.089) (46).

## Expression of ER in Human Cell Lines

Western blotting has been used to detect ERα and ERβ proteins in human urothelial cell lines. ERβ signals were detected in virtually all of the bladder cancer lines examined, while in most of the studies no or very low levels of ERα expression were seen in these lines (19, 39, 42, 47–51). In addition, an immortalized human normal urothelial cell line, SVHUC, was found to express the ERβ protein, but not ERα (48). However, as mentioned above, the specificity of ER antibodies has recently been a critical issue (43, 44). Specifically, there are only a few commercially available antibodies found to be highly specific for ERβ in immunoblotting, such as PPZ0506 (43) and CWK-F12 (44), but such validated ones have not been used in any of the western analyses described above. Moreover, most of these studies lacked adequate positive and/or negative controls.

Quantitative PCR analysis has revealed the status of *ERα*/*ERβ* gene expression in bladder cancer cell lines. The transcripts were detected in all the lines examined in some of the studies (19, 39), while, in others (28, 47, 49), no *ERα* transcript was found in certain cell lines. In addition, a study compared the levels of *ERα* and *ERβ* mRNA expression in human non-neoplastic urothelial cells primarily cultured vs. immortalized and showed higher ERα expression in the latter and similar ERβ levels between them (39).

## Role of ER in Urothelial Tumorigenesis

Carcinogenic compounds, such as N-butyl-N-(4-hydroxybutyl)nitrosamine (BBN) (52) and arsenic (53), have been used to reliably induce bladder cancer in rodents. In the former model, male animals were found to more rapidly develop bladder tumors than females (52, 54).

The efficacy of estrogen in the development of bladder cancer was first reported in 1975 (55). Treatment with a synthetic estrogen diethylstilbestrol in castrated male rats considerably reduced the incidence of BBN-induced bladder tumors, compared with castration only, suggesting the preventive effect of estrogen on bladder tumorigenesis. However, ovariectomy in female rats before (3/10, 30%) or after (3/12, 25%) administration of BBN only slightly promoted the development of bladder tumors (control group: 2/11, 18%). A subsequent study (56) demonstrated that ethinyl estradiol significantly inhibited the occurrence of histologically confirmed bladder carcinoma in BBN-treated male rats (1/27, 4% vs. 10/25, 40%; *P* < 0.01), although the effect of androgen deprivation induced by estrogen administration in males might also have considerably contributed to the inhibition. Meanwhile, tamoxifen, a selective ER modulator, has been shown to strongly prevent BBN-induced carcinogenesis in female mouse bladders (57). Because ERα and ERβ were found to be expressed in none (74% after 12-week exposure to BBN) and all (with or without BBN) of the mouse bladders examined, respectively, the protection effect of tamoxifen was more likely mediated by ERβ. When arsenic was exposed in utero, bladder carcinoma was found only in female mice postnatally treated with diethylstilbestrol (3/33, 9%), but not those with vehicle only (0/34) or tamoxifen (0/35) while benign lesions were developed in a subset of mice (vehicle 15%, tamoxifen 29%, diethylstilbestrol 30%) (58). Immunohistochemistry further showed the enhanced expression of ERα in arsenic/diethylstilbestrol-induced bladder cancer, while ERβ was not examined.

The function of ERα (42) and ERβ (59) in bladder tumorigenesis has further been investigated, using knockout (KO) mouse models along with BBN exposure. In female mice, ERαKO resulted in a significantly (*P* = 0.03) higher incidence of bladder cancer (13/16, 81%), compared with that in wild-type littermates (13/28, 46%). ERαKO males (11/13, 85%) also more frequently developed bladder cancer, compared with wild-type littermates (17/27, 63%), although the difference was not statistically significant. Moreover, similar difference (*P* = 0.0211) was seen between urothelium-specific ERαKO females generated by crossbreeding floxed ERα mice with uroplakin II (UPII) promoter driven Cre transgenic mice (16/21, 76%) versus wild-type females (12/30, 40%) to both of which BBN had been given. By contrast, BBN-induced bladder tumors were more often seen in wild-type males (100%) or females (75%) than in ERβKO males (67%; *P* = 0.2059) or females (23%; *P* = 0.0169). These findings suggest that ERα and ERβ represses and induces, respectively, bladder tumor development.

UPII-SV40T transgenic mice express the simian virus 40 large T antigen specifically in the urothelium and spontaneously develop bladder cancer without sexual bias (60). Using the transgenic model, the volume of bladder tumors was found to be significantly smaller in multiparous animals than in nulliparous females (61), implying a protective role of not only progesterone but also estrogens, both of which are increased during pregnancy (and breastfeeding), in bladder cancer. Indeed, it has been documented that nulliparous women are at a greater risk of bladder cancer development than parous women (62).

An *in vitro* system, using the SVHUC line where the non-neoplastic urothelial cells undergo malignant transformation upon exposure to chemical carcinogens such as 3-methylcholanthrene (MCA) (63), has also been applied to tumorigenesis experiments. ERα overexpression in SVHUC cells resulted in the prevention of MCA-mediated neoplastic transformation, compared with control ERα-negative cells (42). By contrast, ERβ knockdown demonstrated resistance to the neoplastic transformation of MCA-SVHUC cells (59). These findings support *in vivo* data described above indicating inhibitory and stimulatory functions of ERα and ERβ, respectively, in urothelial carcinogenesis. Meanwhile, UDP-glucuronosyltransferase 1A (UGT1A) is a group of phase II drug metabolism enzymes known to prevent from cancer initiation by detoxifying bladder carcinogens such as aromatic amines and metabolites from tobacco (64). In SVHUC cells, E2 (presumably *via* ERβ) has been shown to induce UGT1A expression (48), implying a preventive role of estrogen/ERβ in urothelial tumorigenesis. In line with the effect of estrogen in SVHUC cells, ovariectomy in the female mice significantly reduced the expression levels of *Ugt1a* subtypes in their bladders (48).

## Role of ER in Urothelial Tumor Progression

The effects of ER ligands on urothelial tumor progression have been assessed. An early study demonstrated that the transplantable urothelial tumor did not survive in most of male mice treated with E2 and all of untreated female mice (65). In a case study, complete remission of bladder cancer metastasis was achieved in a male patient following tamoxifen treatment for painful gynecomastia the origin of which was uncertain (66). Subsequent studies in bladder cancer lines expressing ERα showed that E2 induced cell proliferation (28, 39), while ER antagonists, including tamoxifen, raloxifene, and a pure anti-estrogen ICI 182,780, inhibited it (28, 39, 67). In addition, tamoxifen and raloxifene inhibited the growth of bladder cancer cells negative for ERα and positive for ERβ (determined *via* western blot) (19, 47, 67, 68) and xenograft tumors derived from these cells (68). In ERα/ERβ knockdown cells, raloxifene did not significantly affect their growth (67). Overall, estrogens appear to promote the growth of urothelial cancer.

More specifically, both an ERα-selective ligand [*i.e.* propyl pyrazole triol (PPT)] and an ERβ-selective ligand [*i.e.* diatylpropionitrile (DPN)] induced the proliferation of ERα-positive/ERβ-positive bladder cancer cells, but not those expressing ERα-siRNA (for PPT) and ERβ-siRNA (for DPN) (39). Consistent with these data, knockdown of ERβ (49, 59) or treatment with a selective ERβ antagonist (*i.e.* PHTPP) (59) resulted in the suppression of cell growth. ERβ knockdown was also associated with the reduction of cell invasion (59). In contrast to the findings in SVHUC cells, E2 down-regulated the expression of a potential tumor suppressor UGT1A in ERα-negative/ERβ-positive bladder cancer cells (48). Nonetheless, ERα activation was suggested to have an inhibitory role in tumor growth by showing that its knockdown induced the growth of cancer cells and xenograft tumors, while its overexpression showed the opposite effect (42). Moreover, in bladder cancer specimens, UGT1A expression was positively and negatively correlated with that of ERα and ERβ, respectively (48). Thus, there are consistent data for the oncogenic role of ERβ, yet the findings are conflicting as to the function of ERα in urothelial cancer progression. Importantly, the status of ERα expression in some bladder cancer lines (*e.g.* T24) remains controversial (28, 39, 42, 49, 59, 67). In addition, one study showed the suppression of bladder cancer cell migration and invasion by DPN, potentially *via* increasing E-cadherin expression and decreasing N-cadherin expression (31).

Underlying mechanisms for the functions of ERs have further been explored. It was shown that E2 could induce the expression of phospho-ERK in bladder cancer cells (39, 49) and that raloxifene could increase apoptosis *via* inducing the cleavage of caspase-3 and BAD (47, 67). We recently reported that ERβ (via binding to its promoter) inactivated FOXO1, a transcription factor shown to function as a suppressor for urothelial tumor, in bladder cancer cells (69). The link between ERα signaling and INPP4B-mediated AKT activity (42), as well as between ERβ signaling and MCM5 (57) involving the initiation of DNA replication, has also been suggested.

ER signaling has been implicated in the modulation of microRNA (miRNA), circular RNA (circRNA), and enhancer RNA (eRNA) all of which are known to play an important role in bladder cancer progression. ERα has been shown to induce the expression of miR-4324 *via* binding to its promoter in bladder cancer cells and thereby inhibits cell proliferation and metastasis (70). Similarly, ERβ could increase miR-92a expression *via* binding to the promoter of its host gene C13orf25 in bladder cancer cells and promoted cell growth/invasion (71). ERα also reduced circ_0023642 expression by regulating the expression of its host gene UVRAG and subsequently induced miR-490-5p expression, resulting in the down-regulation of EGFR expression and inhibition of bladder cancer cell invasion (72). In addition, knockdown of each estrogen-responsive eRNA, eGREB1 (73) or P2RY2e (74), in bladder cancer lines resulted in the inhibition of cell proliferation/migration/invasion and the induction of apoptosis, suggesting their oncologic role.

Several studies have assessed the involvement of ER signaling in the microenvironment of bladder cancer, such as cancer-associated immune cells and cancer-associated fibroblasts (CAFs) that are known to modulate tumor progression. First, co-culture of CD4+ T cells promoted the proliferation and invasion of bladder cancer cells and considerably increased the expression of not only *MET* oncogene and c-MET but also ERβ in bladder cancer cells (75). Second, ERβ knockdown in co-cultured mast cells resulted in significant reduction in bladder cancer cell invasion *via* modulating epithelial-to-mesenchymal transition and CCL2/CCR2/MMP9 signals (76). Third, co-culture of CAFs induced the growth of bladder cancer cells as well as the mRNA and protein levels of ERβ in bladder cancer cells (51). Finally, overexpression or knockdown of ERα in co-cultured fibroblasts enhanced or reduced, respectively, the fibroblast-induced invasion of bladder cancer cells (77).

In addition to nuclear ERs, membrane ERs that are cell surface receptors and mediate the non-genomic effects of estrogens, including G protein-coupled estrogen receptor 1 (GPER, also known as GPR30), have been identified (78). Inhibition of the estrogen-induced proliferation of bladder cancer cells *via* the GPR30 pathway has indeed been documented (49). An earlier study also showed that GPR30 expression in a bladder cancer line was inversely associated with E2-mediated cell proliferation and c-fos/c-jun/cyclin D1 expression and that G-1, a GPR30-specific agonist, suppressed the cell growth (79). These findings suggest an inhibitory role of GPR30 in urothelial cancer progression.

## Role of ER in Sensitivity to Conventional Non-Surgical Therapy

Cisplatin-based combination chemotherapy, such as MVAC (methotrexate/vinblastine/doxorubicin/cisplatin) and GC (gemcitabine/cisplatin), remains the mainstay of the treatment for locally advanced or metastatic bladder cancer. Doxorubicin, as well as mitomycin C or thiotepa, has also been used for prophylactic intravesical chemotherapy, primarily in patients with superficial bladder tumor following transurethral surgery. In addition to their role in urothelial tumorigenesis and tumor progression, ER signals have been linked to chemosensitivity in bladder cancer.

In bladder cancer lines, tamoxifen, together with methotrexate, vinblastine, doxorubicin, or cisplatin (80), as well as doxorubicin, mitomycin C, or thiotepa (81), was found to more strongly inhibit cell proliferation, compared to that with each cytotoxic agent alone. However, in these assays, the rates of inhibition by tamoxifen in the absence vs. presence of each anti-cancer drug were not directly compared, and it was therefore difficult to assess if tamoxifen could modulate sensitivity to each agent. The same group conducted a clinical study in 30 patients with advanced bladder cancer who were treated with a combination of cisplatin, methotrexate, and vinblastine, and a high dose of tamoxifen (200 mg/m^2^/day, days 1-4), and concluded that the response rate was comparable to conventional cisplatin-based combination chemotherapy (82). However, there was no control arm with chemotherapy alone in this trial. An additional *in vitro* study showed that gemcitabine plus tamoxifen more strongly inhibited the growth of bladder cancer cells and induced apoptosis, compared with gemcitabine or tamoxifen alone (50).

More recent studies have assessed the role of ERβ signaling in modulating sensitivity to cisplatin in bladder cancer. Co-culture of CAFs reduced the cytotoxicity of cisplatin in bladder cancer cells while inducing ERβ expression (51). We recently demonstrated that ERβ knockdown or tamoxifen treatment in ERα-negative bladder cancer cells enhanced sensitivity to cisplatin and that E2 treatment showed the opposite effect (35). Moreover, the considerable induction in ERβ expression was seen in cisplatin-resistant sublines established by long-term culture with low/increasing doses of cisplatin, compared with respective controls. In these cisplatin-resistant cells, E2 treatment was also found to increase the expression and activity of β-catenin known to contribute to cisplatin resistance. We additionally showed that FOXO1, which could be inactivated *via* the ERβ pathway as described above (69), was also inactivated in cisplatin-resistant bladder cancer cells and that FOXO1 knockdown or inhibitor treatment significantly induced resistance to cisplatin in bladder cancer cells (83). Meanwhile, in transurethral resection specimens from those, especially female patients, who subsequently received cisplatin-based neoadjuvant chemotherapy prior to radical cystectomy, loss of ERβ immunoreactivity was strongly associated with favorable chemoresponse (35). Similarly, high ERβ expression in adjacent normal bladder tissues was strongly associated with worse patient outcomes after cisplatin-based chemotherapy (51). Additionally, in a study showing the induction of miR-4324 expression by ERα (70), bladder cancer sublines stably expressing miR-4324 were found to be significantly more sensitive to doxorubicin treatment than respective control sublines. These findings suggest that activation of ERα or ERβ is associated with increased sensitivity to doxorubicin treatment or resistance to cisplatin treatment, respectively, in bladder cancer.

The impact of ER signaling on the efficacy of BCG immunotherapy often used for the treatment of non-muscle-invasive bladder cancer has also been investigated. Indeed, as aforementioned, female bladder cancer patients have lower response rates to intravesical BCG therapy (5). In bladder cancer cells expressing both ERα and ERβ, E2 reduced BCG internalization, while tamoxifen and ICI 182,780 induced it (84). These antagonists were also found to enhance the effect of BCG both *in vitro* and *in vivo* (84), suggesting that targeting ER signals *via* anti-estrogen treatment during BCG therapy might be a useful sensitization strategy. In addition, because ER activation has been suggested to result in the reduction of sensitivity to newly-developed immunotherapy with inhibitors of programmed cell death-1 (PD-1) or its ligand (PD-L1) in, for instance, breast cancer (85), it would be interesting to assess if this can be observed in urothelial cancer.

## Clinical Trials of ER Modulation in Bladder Cancer

Two phase 2 trials involving bladder cancer in relation to ER signaling appear to be ongoing. One is to investigate the efficacy of tamoxifen in non-muscle-invasive disease (NCT02197897). In these patients with low- to intermediate-risk disease, 12-week treatment with a single daily oral dose of 20 mg is being tested for the clinical response, along with the immunohistochemical assessment of several markers including ERα and ERβ, in the post-treatment biopsy specimens. The other is to determine whether genistein, a biologically active isoflavone and a phytoestrogen with structure similar to that of E2 (86), not only reduces the adverse effects of intravesical BCG therapy but also improves its efficacy (NCT01489813). In these patients with superficial disease, 30 mg of genistein is being administered orally 3 times daily for 10 weeks (during BCG therapy and one-month post-therapy), and the changes in severity of urinary symptoms as well as the rates of tumor recurrence are compared with those who receive placebo pills. In another phase 2 study (NCT00710970) completed in 2012, the efficacy of tamoxifen was assessed in a total of 28 patients with metastatic bladder cancer who had undergone chemotherapy. However, no results from these studies have been reported.

Further clinical studies are thus required for determining the actual benefit of anti-estrogen (or estrogen) treatment in patients with urothelial cancer. In particular, more convincing preclinical data exist to predict enhanced sensitivity to conventional non-surgical therapy against bladder cancer *via* anti-estrogens (35, 50, 70, 80, 81, 83). In these studies, the role of ERα/ERβ expression in clinical samples, as a predictor of therapeutic response, may also need to be explored. In addition, to facilitate the conduction of clinical trials, it is of importance to assess the functional activity, instead of the expression status, of ERα/ERβ in surgical specimens and its association with patient outcomes.

## Conclusions

Current evidence indicates a critical role of estrogen-mediated ER signaling in the pathogenesis of urothelial cancer. This further supports that urothelial cancer is an endocrine-related neoplasm. Specifically, various studies have suggested that ERα and ERβ play protective and oncogenic roles, respectively, in urothelial tumorigenesis and tumor progression. Thus, the stimulatory and inhibitory effects of estrogens appear to be dependent on the functional activity of ERα versus ERβ in each tumor. Nonetheless, conflicting results, especially those on ERα functions, exist, implying that the actions of estrogens can even be cell-specific. Moreover, the prognostic significance of ERα/ERβ expression in urothelial cancer specimens remains controversial. The impact of ER signaling on urothelial cancer might thus be context-dependent. Notably, the specificity of commercially available anti-ER antibodies, especially those against ERβ, has been regarded as a critical issue, which may make many of previous studies ineligible. Further investigation of ERs, as well as other molecules directly or indirectly regulated by estrogens, along with the use of validated antibodies, is thus required for determining the precise actions of estrogens in urothelial cells and their underlying molecular mechanisms.

## Author Contributions

TG and HM have equally contributed to the literature review. TG has drafted and HM has finalized the manuscript. All authors contributed to the article and approved the submitted version.

## Conflict of Interest

HM has received research funding from Astellas Scientific and Medical Affairs, Ferring Research Institute, and Bristol-Myers Squibb.

The remaining author declares that the research was conducted in the absence of any commercial or financial relationships that could be construed as a potential conflict of interest.
